# Impact of albumin infusion on prognosis in ICU patients with cirrhosis and AKI: insights from the MIMIC-IV database

**DOI:** 10.3389/fphar.2024.1467752

**Published:** 2024-10-07

**Authors:** Mengqi Li, Yidi Ge, Jingjing Wang, Wenya Chen, Jiashuo Li, You Deng, Wen Xie

**Affiliations:** ^1^ Center of Liver Diseases, Beijing Ditan Hospital, Capital Medical University, Beijing, China; ^2^ Liver Research Center, Beijing Friendship Hospital, Capital Medical University, Beijing, China; ^3^ Beijing Key Laboratory of Translational Medicine on Liver Cirrhosis, Beijing Friendship Hospital, Capital Medical University, Beijing, China; ^4^ National Clinical Research Center for Digestive Diseases, Beijing Friendship Hospital, Capital Medical University, Beijing, China

**Keywords:** cirrhosis, acute kidney injury, albumin therapy, 28-day mortality, inverse probability of treatment weighting

## Abstract

**Background:**

Acute kidney injury (AKI) is common in cirrhotic patients, especially in the intensive care unit (ICU), and is often associated with poor prognosis. Albumin is often used for plasma volume expansion, but its efficacy in cirrhotic patients with AKI [excluding hepatorenal syndrome (HRS)] is debated. This study aimed to assess the impact of albumin therapy on prognosis in ICU patients with cirrhosis and non-HRS AKI.

**Methods:**

A retrospective analysis was conducted using the MIMIC-IV 2.2 database. The primary endpoint was 28-day mortality. Inverse probability of treatment weighting (IPTW) was used to balance baseline characteristics between the albumin and non-albumin groups.

**Results:**

A total of 1,623 patients were included, with 586 receiving albumin. After IPTW, the sample sizes were 1,713 in the non-albumin group and 1,490 in the albumin group. Albumin administration was associated with higher rates of AKI recovery at 48 h but did not improve 28-day mortality in the overall cohort. Further analysis revealed that using 5% albumin concentration was associated with improved 28-day mortality (HR 0.68; 95% CI 0.49–0.95; *p* = 0.025), whereas 25% albumin did not show benefit. In patients with high bilirubin levels, albumin treatment significantly reduced 28-day mortality. However, albumin therapy may increase 28-day mortality in certain subgroups, including patients with chronic kidney disease and baseline albumin levels >3.3 g/dL.

**Conclusion:**

Although albumin therapy improved 28-day mortality in some cases, it may also increase mortality in certain subgroups. The use of albumin in critically ill patients with cirrhosis and AKI should be approached with greater consideration of its risks and benefits.

## 1 Introduction

Acute kidney injury (AKI) is a prevalent condition in patients with cirrhosis, affecting up to 82.5% of those in the intensive care unit (ICU) (Amathieu et al., 2017). The presence of AKI in cirrhosis is linked to a poor prognosis (Desai et al., 2020). The most common etiology was prerenal AKI, followed by acute tubular necrosis (Patidar et al., 2023).

Albumin is the first-line plasma expander for hospitalized patients with cirrhosis and AKI (European Association for the Study of the LiverEuropean Association for the Study of the Liver, 2018). It is more effective than the saline solution in restoring effective arterial blood volume in patients with cirrhosis and ascites (Garcia-Tsao et al., 2024). Besides maintaining osmotic pressure, albumin also exerts multiple functions such as antioxidation, inhibition of systemic inflammation, immunomodulation, and endothelial stabilization (Garcia-Martinez et al., 2013), potentially providing additional beneficial effects. Moreover, when combined with splanchnic vasoconstrictors, albumin effectively improves kidney function in patients with hepatorenal syndrome (HRS) (Wong et al., 2021). These findings have led to albumin being recommended in guidelines as the treatment for AKI in patients with cirrhosis. However, current research primarily focuses on HRS-AKI, which is relatively uncommon (Patidar et al., 2023). More attention is needed for non-HRS AKI, as it represents a larger proportion of cases.

Currently, the benefit of albumin infusion in cirrhosis patients with non-HRS AKI remains controversial (Patidar et al., 2022; Giri et al., 2022). A previous study has shown that albumin use in patients with non-HRS AKI did not improve AKI recovery or in-hospital mortality (Patidar et al., 2022). However, this study excluded ICU patients. ICU patients are unique, as they may have more severe hemodynamic instability, shock, or respiratory failure. These patients may require more aggressive treatment. However, considering the potential side effects of albumin, such as pulmonary edema or fluid overload (Maiwall et al., 2022; China et al., 2021), it is unclear whether the benefits of albumin infusion outweigh the risks for this population. Currently, no research has explored whether albumin therapy can improve outcomes in ICU patients with cirrhosis and non-HRS AKI. Therefore, this study focuses on ICU patients with cirrhosis and non-HRS AKI to explore the impact of albumin infusion on patient outcomes.

## 2 Patients and methods

### 2.1 Study population

A retrospective analysis was conducted using the Medical Information Mart for Intensive Care IV (MIMIC-IV) 2.2 database. This database includes comprehensive information on patients admitted to the ICUs at Beth Israel Deaconess Medical Center (BIDMC) from 2008 to 2019 (Johnson et al., 2023). The Institutional Review Board of BIDMC granted a waiver of informed consent and approved resource sharing, as all patients in the database were de-identified for privacy protection. Before data extraction, the author Mengqi Li obtained access to the database and was responsible for the data extraction.

**FIGURE 1:**
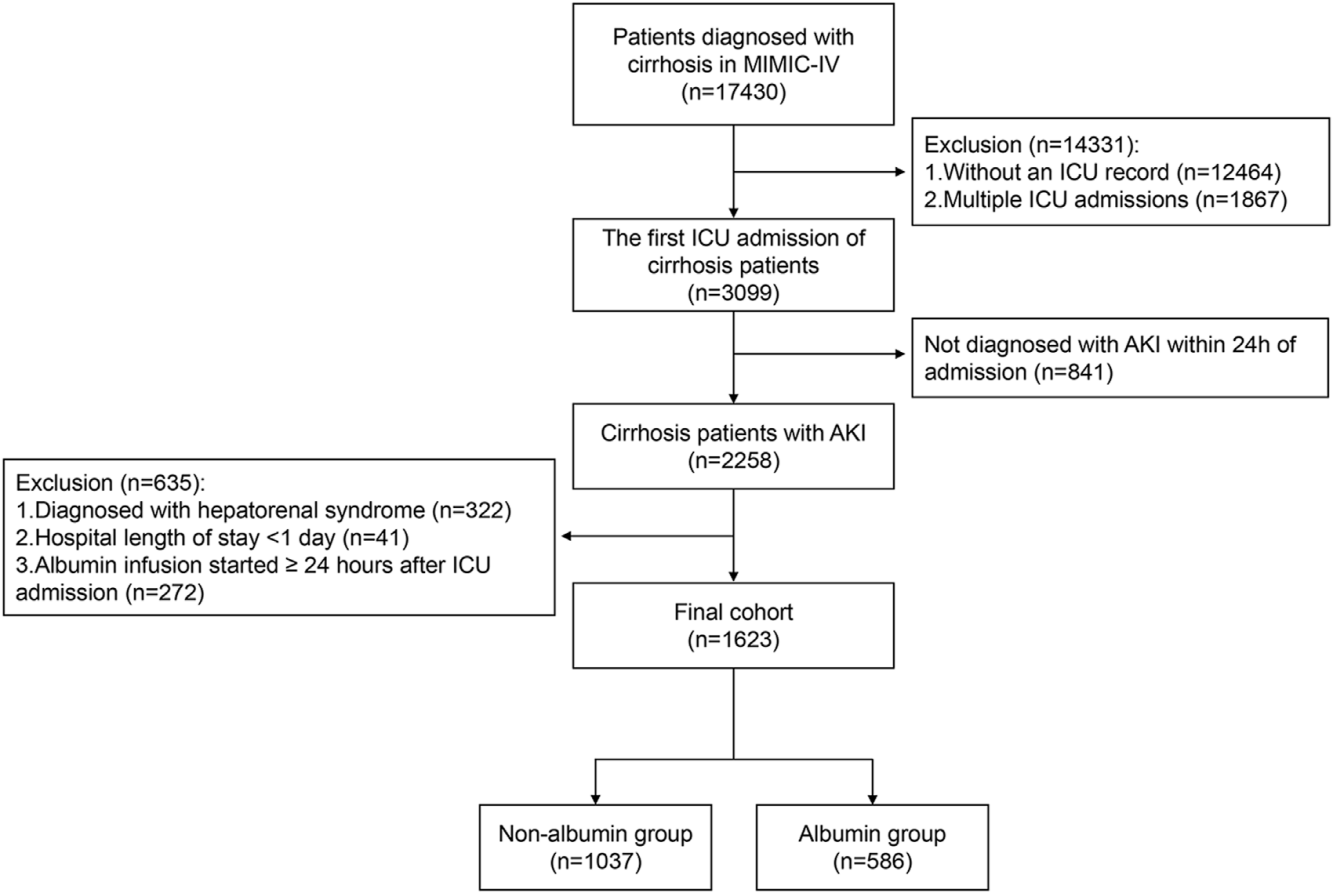
Flow chart of the study participants. Abbreviations: MIMIC-IV, Medical Information Mart for Intensive Care IV; ICU, intensive care unit; AKI, acute kidney injury.

The study included patients diagnosed with liver cirrhosis who were admitted to the ICU. For patients with multiple ICU admissions, only the first ICU stay was considered. The exclusion criteria were as follows: (1) patients not diagnosed with AKI within 24 h of admission (n = 841); (2) patients diagnosed with HRS (n = 322); (3) patients with a hospital stay of less than 24 h (n = 41); (4) patients who began albumin use 24 h or more after ICU admission (n = 272). The flowchart for patient enrollment is presented in Figure 1.

### 2.2 Data collection and definitions

Medical data of cirrhotic patients in the MIMIC-IV 2.2 database were extracted using Navicat Premium software (version 16.1.12) through the execution of Structured Query Language queries. Extracted variables included demographic information, laboratory parameters, vital signs, clinical severity scores, total urine output, treatments (such as the use of albumin, diuretics, etc.) during the first 24 h after ICU admission, related complications or comorbidities, and prognosis information. For laboratory indicators measured multiple times, we selected the maximum or minimum values based on clinical significance, such as the maximum bilirubin or minimum albumin levels.

The use of albumin was initially defined as the infusion of albumin within the first 24 h of ICU admission. The diagnosis and etiology of cirrhosis, along with information on cirrhosis-related complications (including ascites, esophageal variceal hemorrhage, hepatic encephalopathy, and spontaneous bacterial peritonitis), were extracted using inpatient ICD-9 and ICD-10 codes (Supplementary Table S1). Acute kidney injury and its stages were defined based on the Kidney Disease: Improving Global Outcomes Clinical Practice Guidelines (Kellum et al., 2013). Full recovery from AKI was defined as the absence of AKI criteria at 48 h after ICU admission (Forni et al., 2017). Regression of AKI was defined as a decrease in AKI stage at 48 h after ICU admission (Forni et al., 2017). The primary endpoint was 28-day mortality, while secondary endpoints included ICU mortality, full recovery from AKI, and regression of AKI at 48 h after ICU admission.

### 2.3 Statistical analysis

Categorical data were expressed as frequencies (percentages) and continuous data as median (interquartile range). Continuous variables were compared using the Mann-Whitney U test. Categorical variables were compared using the chi-square test or Fisher’s exact test, as appropriate. For paired samples, the Wilcoxon matched-pair signed test were used. Multiple imputation was conducted to account for the missing data (Pedersen et al., 2017). Specific details of variable missingness are provided in Supplementary Table S2.

Inverse probability of treatment weighting (IPTW) was conducted to balance the baseline characteristics between the albumin group and the non-albumin group. Propensity scores were calculated using multiple logistic regression adjusted for demographics (age, gender), etiology of cirrhosis, biochemical indicators (creatinine, albumin, total bilirubin, transaminases, etc.), coagulation indicators, routine blood tests, and baseline complications. Covariate balance before and after IPTW was assessed using the standardized mean difference (SMD). A variable was considered balanced between the groups when its SMD was <0.2 (Cohen, 2013). Weights based on propensity scores were recalculated for each subgroup analysis.

Univariate and multivariate Cox regression analyses were performed to identify risk factors associated with prognosis. A two-sided *p*-value <0.05 was considered statistically significant. All statistical analyses were performed with R version 4.2.2 (R Foundation for Statistical Computing, Vienna, Austria).

## 3 Results

### 3.1 Albumin administration in the study

The MIMIC-IV database included 3,099 patients with cirrhosis, among whom 2,258 were diagnosed with acute kidney injury within 24 h of ICU admission. Finally, 1,623 patients who met the inclusion criteria were enrolled in the study. Of these, 586 patients (36.1%) received albumin treatment within the first 24 h of ICU admission, while 1,037 patients (63.9%) did not receive albumin treatment (Figure 1). Despite not receiving albumin, 993 of the 1,037 patients (95.8%) received crystalloid therapy.

**FIGURE 2:**
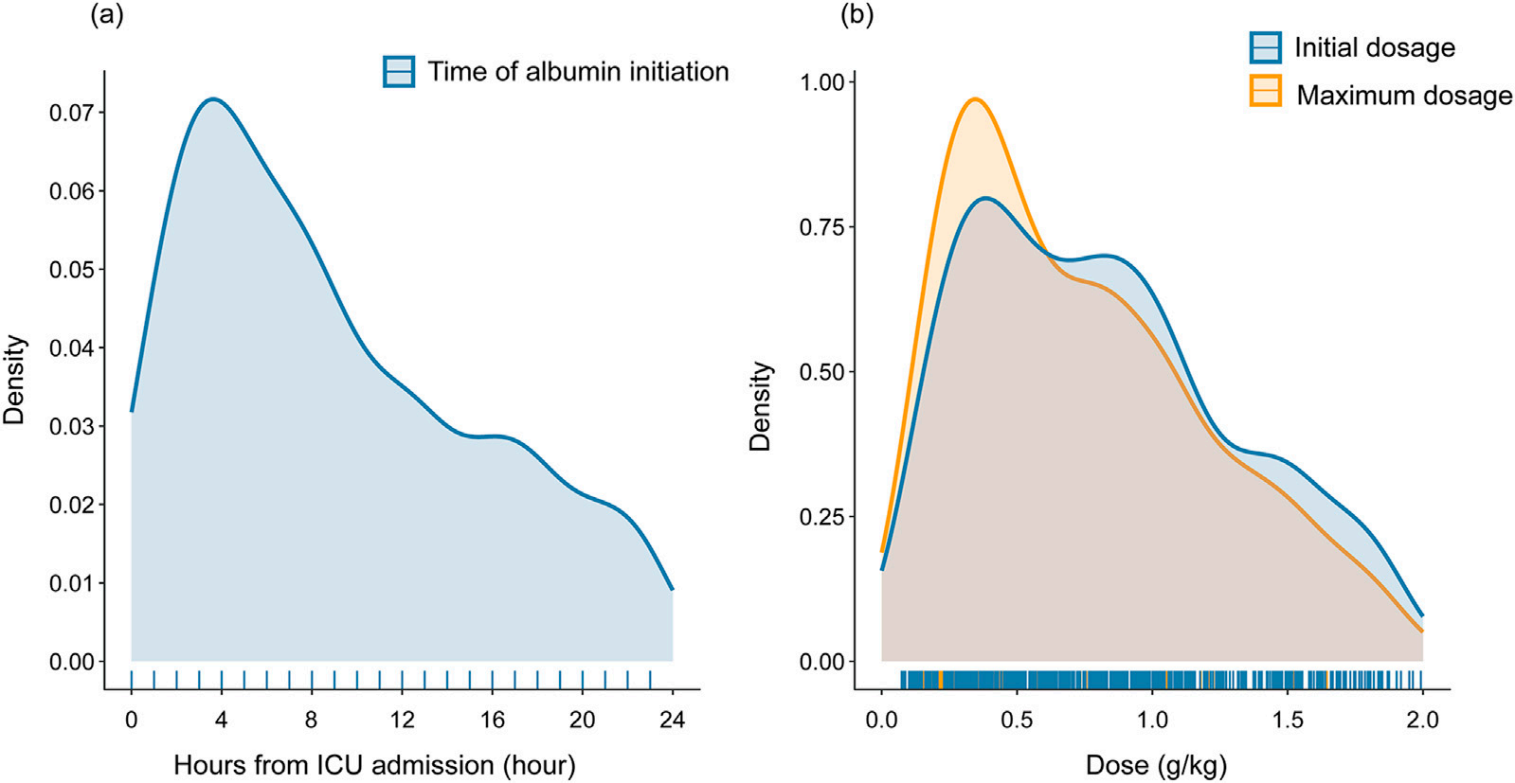
Distribution of time to initiate albumin administration **(A)** and dosage of albumin therapy **(B)**. Abbreviations: ICU: intensive care unit.

The median time to initiate albumin administration from ICU admission was 7 h (interquartile range: 4–13). The initial and maximum daily doses were 0.69 g/kg (interquartile range: 0.35–1.11) and 0.81 g/kg (interquartile range: 0.43–1.25), respectively. The median duration of albumin treatment was 1 day (interquartile range: 1–4 days). Among the 586 patients who received albumin, the average total dose over 28 days was 1.7 g/kg. For the 293 patients who received albumin for more than 1 day, the average total dose was 2.6 g/kg over the 28-day period. Additionally, 374 patients received a 25% albumin solution, while 212 patients received a 5% albumin solution. Figure 2 illustrates the time distribution from ICU admission to the start of albumin administration (Figure 2A), as well as the initial and maximum daily doses of albumin therapy (Figure 2B).

### 3.2 Baseline characteristics of patients with and without albumin infusion before and after IPTW

Compared to the non-albumin group, the albumin group had a higher proportion of females and exhibited more severe illness (Table 1, *p* < 0.05). Specifically, they showed greater liver dysfunction, indicated by elevated levels of transaminases, bilirubin, international normalized ratio, and lower albumin levels (Table 1, *p* < 0.05). They also had more severe kidney dysfunction, with higher proportions of AKI stages 2 and 3, and higher rates of comorbidities and cirrhosis-related complications (Table 1, *p* < 0.05). Albumin administration significantly increased serum albumin levels, while levels decreased in patients who did not receive albumin (Supplementary Table S3, *p* < 0.05). Notably, the albumin treatment group had higher rates of 28-day mortality (17.1% vs. 32.9%, *p* < 0.05) and ICU mortality (8.3% vs. 19.5%, *p* < 0.05).

**TABLE 1:** Baseline characteristics of patients with and without albumin infusion before and after IPTW.

	Before weighting				After weighting			
	Non-albumin group (n = 1,037)	Albumin group (n = 586)	SMD	*p*-value	Non-albumin group (n = 1713)	Albumin group (n = 1,490)	SMD	*p*-value
Age, [years, M(IQR)]	62 (55–70)	61 (54–69)	0.082	0.146	62 (54–70)	62 (55–70)	0.013	0.797
Gender, n (%)			0.105	0.046			0.021	0.779
Male	710 (68.5)	372 (63.5)			1,130 (66.0)	968 (65.0)		
Female	327 (31.5)	214 (36.5)			583 (34.0)	522 (35.0)		
Ethnicity, n (%)			0.052	0.797			0.042	0.939
White	696 (67.1)	394 (67.2)			1,122 (65.5)	998 (67.0)		
Black	91 (8.8)	44 (7.5)			139 (8.1)	125 (8.4)		
Asian	26 (2.5)	14 (2.4)			43 (2.5)	37 (2.5)		
Other	224 (21.6)	134 (22.9)			409 (23.9)	330 (22.1)		
Aetiology of cirrhosis, n (%)			0.219	<0.001			0.136	0.248
Alcoholic only	329 (31.7)	243 (41.5)			579 (33.8)	555 (37.2)		
Viral hepatitis only	228 (22.0)	109 (18.6)			330 (19.3)	331 (22.2)		
Alcoholic + viral	112 (10.8)	67 (11.4)			184 (10.7)	152 (10.2)		
Other	368 (35.5)	167 (28.5)			620 (36.2)	452 (30.3)		
Stage of AKI, n (%)			0.328	<0.001			0.056	0.757
1	343 (33.1)	115 (19.6)		<0.001	468 (27.3)	416 (27.9)		0.841
2	558 (53.8)	354 (60.4)		0.012	942 (55.0)	842 (56.5)		0.685
3	136 (13.1)	117 (20.0)		<0.001	303 (17.7)	233 (15.6)		0.518
Severity of illness								
MELD-Na score, [M(IQR)]	20 (14–30)	29 (20–39)	0.625	<0.001	24 (15–36)	25 (18–34)	0.048	0.357
SOFA score, [M(IQR)]	6 (4–8)	8 (6–11)	0.728	<0.001	7 (5–11)	7 (5–10)	0.029	0.457
GCS, [M(IQR)]	15 (14–15)	15 (14–15)	0.123	0.018	15 (14–15)	15 (14–15)	0.131	0.643
Charlson comorbidity index, [M(IQR)]	6 (4–8)	6 (4–8)	0.122	0.053	6 (4–9)	6 (4–8)	0.040	0.545
Laboratory measurements								
ALT, [U/L, M(IQR)]	38 (22–81)	42 (23–102)	0.090	0.023	37 (22–81)	38 (21–98)	0.032	0.454
AST, [U/L, M(IQR)]	72 (41–160)	101 (50–289)	0.107	<0.001	78 (43–176)	84 (47–228)	0.007	0.079
ALP, [U/L, M(IQR)]	104 (74–152)	109 (76–155)	0.005	0.266	104 (71–153)	107 (74–148)	0.036	0.802
Creatinine, [mg/dL, M(IQR)]	1.2 (0.8–2.0)	1.5 (1.0–2.4)	0.056	<0.001	1.3 (0.9–2.4)	1.5 (1.0–2.1)	0.070	0.163
BUN, [mmol/L, M(IQR)]	26 (17–42)	31 (19–50)	0.175	<0.001	28 (18–50)	29 (19–48)	0.020	0.499
Bicarbonate, [mmol/L, M(IQR)]	21 (18–24)	19 (16–22)	0.400	<0.001	20 (17–23)	20 (17–22)	0.072	0.109
Albumin, [g/dL M(IQR)]	3.0 (2.6–3.4)	2.7 (2.3–3.1)	0.433	<0.001	2.9 (2.5–3.3)	2.8 (2.4–3.2)	0.106	0.053
Total bilirubin, [mg/dL, M(IQR)]	1.9 (1.0–4.4)	3.7 (1.9–9.0)	0.431	<0.001	2.6 (1.2–6.4)	2.8 (1.5–6.0)	0.043	0.485
Chloride, [mmol/L, M(IQR)]	102 (97–106)	101 (95–105)	0.230	<0.001	102 (96–105)	102 (97–105)	0.032	0.830
Sodium, [mmol/L, M(IQR)]	136 (133–139)	135 (131–138)	0.283	<0.001	136 (131–139)	136 (132–138)	0.006	0.814
Potassium, [mmol/L, M(IQR)]	3.9 (3.5–4.3)	3.9 (3.5–4.3)	0.009	0.901	3.9 (3.5–4.4)	3.9 (3.5–4.3)	0.002	0.884
WBC, [10^9^/L, M(IQR)]	10.0 (6.8–14.5)	13.4 (8.8–18.9)	0.360	<0.001	10.9 (7.2–16.6)	11.8 (7.6–17.4)	0.124	0.103
Hemoglobin, [g/dL, M(IQR)]	9.1 (7.8–10.9)	8.3 (7.3–9.6)	0.386	<0.001	8.7 (7.3–10.5)	8.7 (7.4–10.2)	0.007	0.905
Platelet, [10^9^/L, M(IQR)]	93 (60–149)	82 (51–122)	0.215	<0.001	83 (52–137)	89 (53–135)	0.015	0.731
INR, [M(IQR)]	1.6 (1.3–2.0)	1.9 (1.6–2.5)	0.364	<0.001	1.7 (1.4–2.3)	1.8 (1.4–2.3)	0.037	0.547
Lactate, [mmol/L, M(IQR)]	2.4 (1.7–4.0)	3.5 (2.2–5.7)	0.391	<0.001	2.8 (1.8–5.0)	3.1 (2.1–5.0)	0.002	0.148
SpO_2_, [mmHg, M(IQR)]	93 (90–95)	93 (90–95)	0.009	0.275	93 (90–95)	93 (90–95)	0.018	0.933
Vital signs								
Heart rate, [beats/min, M(IQR)]	102 (89–116)	106 (94–120)	0.189	<0.001	104 (89–118)	105 (93–118)	0.079	0.212
MAP, [mmHg, M(IQR)]	59 (51–66)	55 (49–61)	0.344	<0.001	57 (48–64)	56 (50–61)	0.052	0.493
RR, [breaths/min, M(IQR)]	26 (23–31)	28 (24–32)	0.141	<0.001	27 (23–32)	28 (24–32)	0.014	0.581
Temperature, [°C, M(IQR)]	37.2 (36.9–37.6)	37.2 (36.9–37.6)	0.009	0.388	37.2 (36.9–37.6)	37.2 (36.9–37.6)	0.036	0.854
Complications or comorbidities								
Ascites, n (%)	137 (13.2)	174 (29.7)	0.41	<0.001	328 (19.1)	319 (21.4)	0.056	0.391
Esophageal varices with bleeding, n (%)	108 (10.4)	45 (7.7)	0.095	0.085	150 (8.8)	123 (8.3)	0.019	0.776
Hepatic encephalopathy, n (%)	143 (13.8)	125 (21.3)	0.199	<0.001	289 (16.8)	273 (18.3)	0.039	0.559
Spontaneous bacterial peritonitis, n (%)	28 (2.7)	86 (14.7)	0.435	<0.001	177 (10.3)	118 (7.9)	0.084	0.776
Bacterial infections, n (%)	295 (28.4)	205 (35.0)	0.141	0.007	494 (28.8)	465 (31.2)	0.052	0.428
Septic, n (%)	247 (23.8)	140 (23.9)	0.002	1.000	415 (24.2)	341 (22.9)	0.032	0.629
Shock, n (%)	268 (25.8)	307 (52.4)	0.565	<0.001	675 (39.4)	589 (39.5)	0.003	0.970
Treatments within 24 h after ICU admission								
RRT, n (%)	84 (8.1)	43 (7.3)	0.029	0.650	144 (8.4)	117 (7.9)	0.019	0.783
Diuretics, n (%)	252 (24.3)	130 (22.2)	0.050	0.366	381 (22.2)	353 (23.7)	0.034	0.599
NSBB, n (%)	68 (6.6)	27 (4.6)	0.085	0.134	84 (4.9)	82 (5.5)	0.027	0.689
Mechanical ventilation, n (%)	413 (39.8)	325 (55.5)	0.317	<0.001	792 (46.2)	762 (51.1)	0.097	0.177
Urine output, [mL, M(IQR)]	1,250 (750–1880)	940 (510–1,499)	0.425	<0.001	1,095 (560–1,685)	1,123 (644–1741)	0.037	0.624

Abbreviations: IPTW, inverse probability of treatment weighting; SMD, standardized mean difference; AKI, acute kidney injury; MELD-Na, Model for End-Stage Liver Disease-Sodium; SOFA, sequential organ failure assessment; GCS, glasgow coma scale; ALT, alanine aminotransferase; AST, aspartate aminotransferase; ALP, alkaline phosphatase; BUN, blood urea nitrogen; WBC, white blood cells; INR, international normalized ratio; SpO_2_, peripheral capillary oxygen saturation; MAP, mean arterial pressure; RR, respiration rate; ICU, intensive care unit; RRT, renal replacement therapy; NSBB, non-selective beta blocker.

IPTW was performed to balance differences in baseline characteristics between the two groups. After IPTW, the sample sizes were 1713 in the non-albumin group and 1,490 in the albumin group. All baseline characteristics were comparable between the two groups in the IPTW cohort, with SMD less than 0.2 and all *p*-values greater than 0.05 (Table 1).

### 3.3 Impact of albumin treatment on outcomes in the IPTW cohort

**FIGURE 3:**
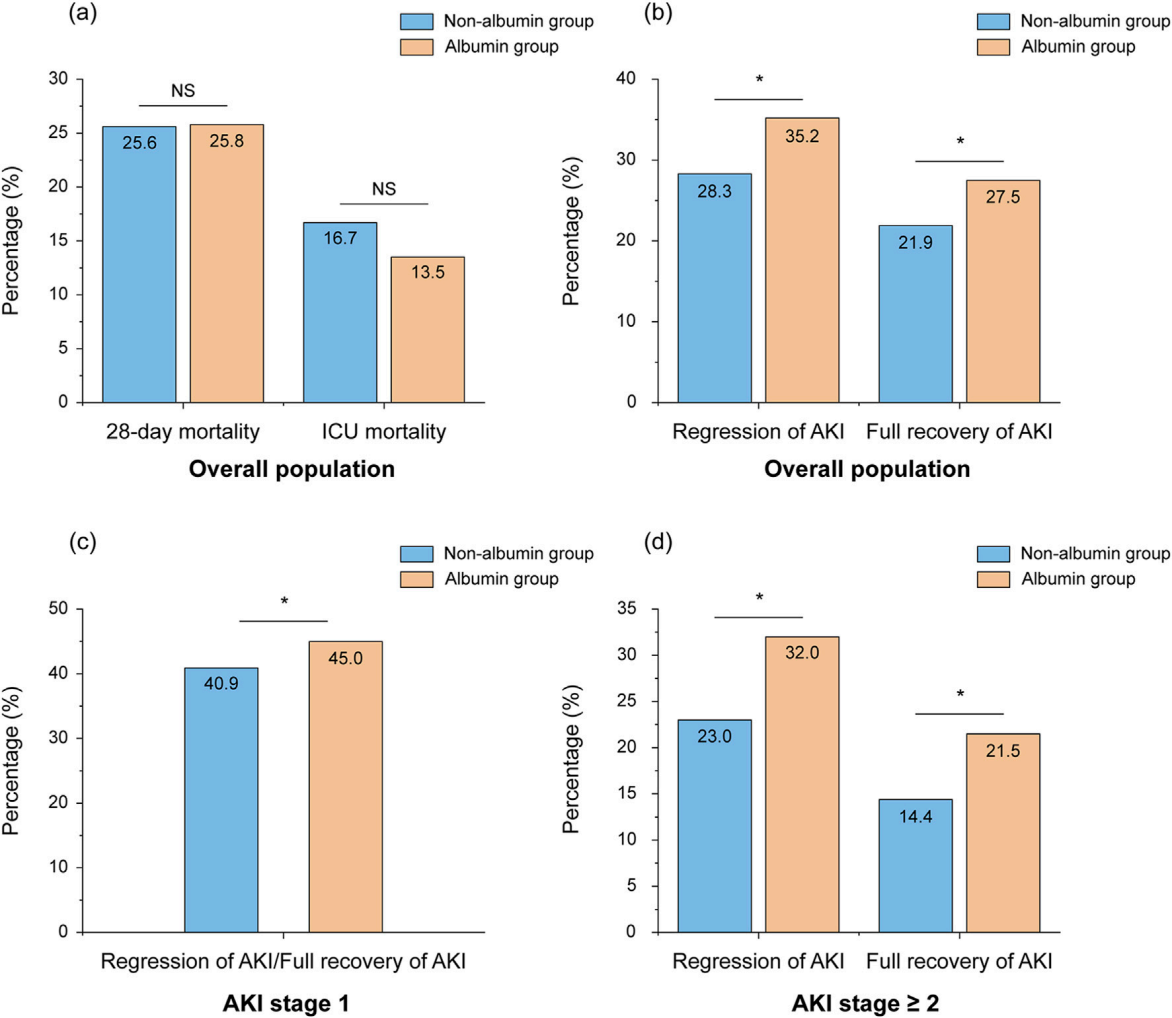
Percentage of mortality **(A)** and regression/full recovery of AKI **(B–D)** in patients with and without albumin infusion after weighting. *Stands for *p*-value < 0.05, NS stands for nonsignificant. Abbreviations: NS, nonsignificant; ICU, intensive care unit; AKI, acute kidney injury.

In the matched cohort, the albumin group showed significantly higher rates of AKI regression and full recovery within 48 h of ICU admission compared to the non-albumin group (Figure 3, *p* < 0.05). This difference was especially notable in patients with AKI stage ≥2 (Figure 3, *p* < 0.05). Although ICU mortality was lower in the albumin group compared to the non-albumin group, the difference was not statistically significant (16.7% vs. 13.5%, *p* > 0.05). Furthermore, no significant difference was observed in 28-day mortality between the two groups (Figure 3).

### 3.4 Impact of albumin treatment on outcomes in different subgroups

**FIGURE 4:**
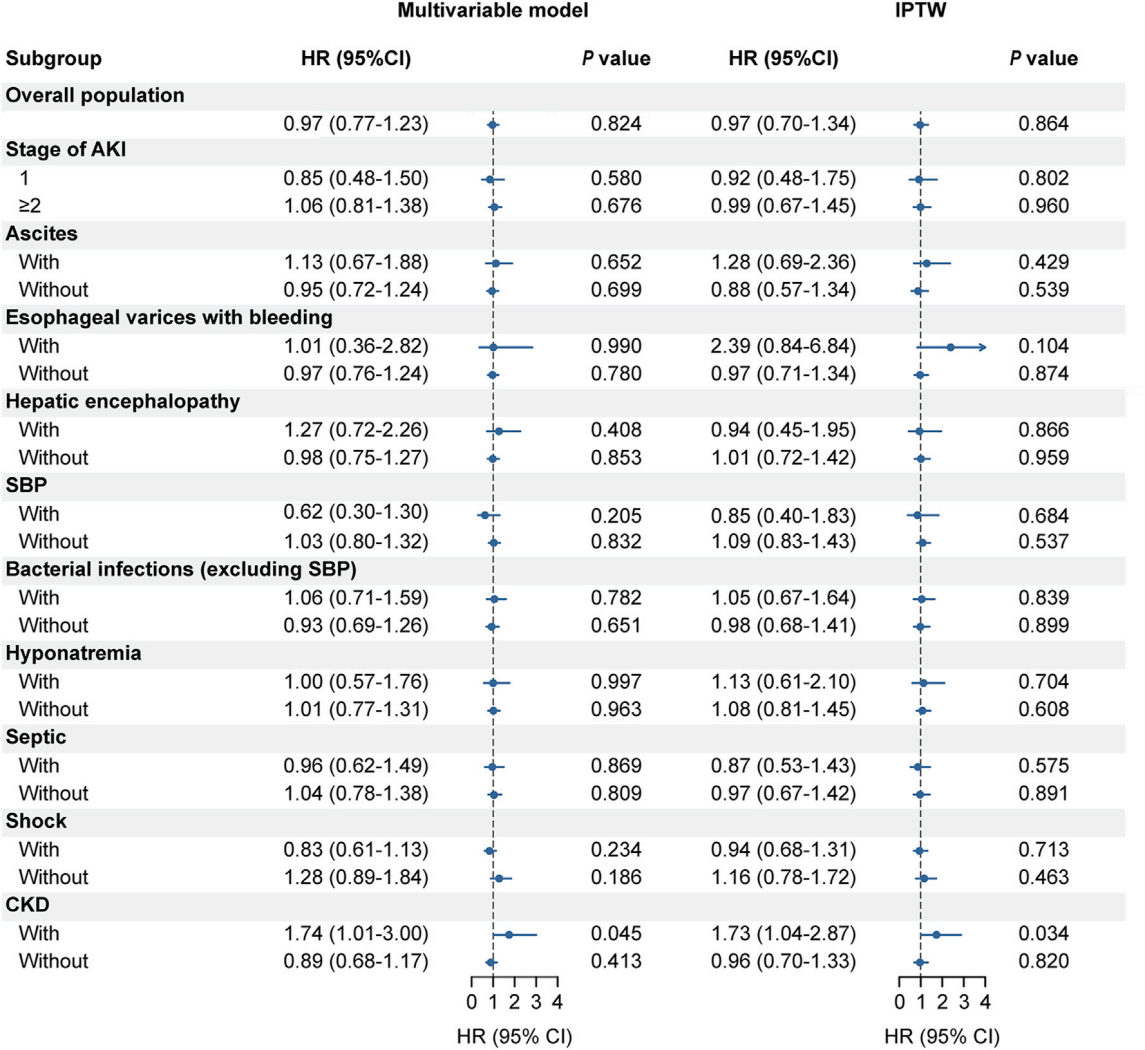
Subgroup analyses of the association between albumin treatment and 28-day mortality based on complications or comorbidities. The multivariate model was adjusted for age, ethnicity, total bilirubin, creatinine, urine output, INR, sodium, albumin, WBC, hemoglobin, lactate, SpO_2_, SOFA score, ascites, hepatic encephalopathy, and SBP. Abbreviations: IPTW, inverse probability of treatment weighting; HR, hazard ratio; AKI, acute kidney injury; SBP, spontaneous bacterial peritonitis; CKD, chronic kidney disease; INR, international normalized ratio; WBC, white blood cells; SpO_2_, peripheral capillary oxygen saturation; SOFA, Sequential Organ Failure Assessment.

In the subgroup analysis, we found that albumin treatment did not improve 28-day mortality across different AKI stages or among various related comorbidities and complications. Both multivariable Cox regression and IPTW-adjusted Cox regression analyses confirmed these findings (Figure 4, *p* > 0.05). Notably, in patients with pre-existing chronic kidney disease (CKD), albumin use was associated with worse outcomes, increasing the likelihood of 28-day mortality (Figure 4, *p* < 0.05).

We further stratified patients based on the dosage, concentration, and duration of albumin use. Results showed no improvement in 28-day mortality with either low- or high-dose albumin, or with varying durations of treatment, compared to no albumin use (Table 2, *p* > 0.05). However, a 5% albumin concentration was associated with improved patient outcomes compared to no albumin use (Table 2, HR 0.68; 95% CI 0.49–0.95; *p* = 0.025), while a 25% concentration did not show any benefit.

**TABLE 2:** Subgroup analyses of the association between albumin treatment and 28-day mortality.

Subgroup	Univariable model		Multivariable model	
	HR (95% CI)	*p*-Value	HR (95% CI)	*p*-Value
Albumin-dosage				
Non-albumin treatment (n = 1,037)	Ref		Ref	
<1.0 g/kg/day (n = 398)	0.59 (0.12–4.88)	<0.001	0.89 (0.69–1.16)	0.389
≥1.0 g/kg/day (n = 188)	1.06 (0.14–7.84)	<0.001	1.17 (0.86–1.59)	0.324
Albumin-dosage				
Non-albumin treatment (n = 1,037)	Ref		Ref	
<1.5 g/kg/day (n = 513)	1.92 (1.55–2.38)	<0.001	0.94 (0.73–1.19)	0.589
≥1.5 g/kg/day (n = 73)	3.83 (2.70–5.44)	<0.001	1.26 (0.84–1.89)	0.266
Duration of albumin use				
Non-albumin treatment (n = 1,037)	Ref		Ref	
<2 days (n = 293)	1.98 (1.53–2.55)	<0.001	1.04 (0.79–1.38)	0.777
≥2 days (n = 293)	2.27 (1.78–2.88)	<0.001	0.92 (0.70–1.21)	0.540
Duration of albumin use				
Non-albumin treatment (n = 1,037)	Ref		Ref	
<4 days (n = 478)	2.05 (1.65–2.55)	<0.001	1.00 (0.78–1.28)	0.984
≥4 days (n = 108)	2.42 (1.74–3.38)	<0.001	0.87 (0.61–1.26)	0.472
Albumin solution type				
Non-albumin treatment (n = 1,037)	Ref		Ref	
25% (n = 374)	2.46 (1.97–3.08)	<0.001	1.19 (0.92–1.54)	0.192
5% (n = 212)	1.58 (1.17–2.14)	0.003	0.68 (0.49–0.95)	0.025

Multivariate model was adjusted for age, ethnicity, total bilirubin, creatinine, urine output, INR, sodium, albumin; WBC, hemoglobin, lactate, SpO_2_, SOFA, score, ascites, hepatic encephalopathy, and SBP.

Abbreviations: HR, hazard ratio; INR, international normalized ratio; WBC, white blood cells; SpO_2_, peripheral capillary oxygen saturation; SOFA, sequential organ failure assessment; SBP, spontaneous bacterial peritonitis.

Not all patients stopped using diuretics or nephrotoxic drugs upon admission, and stratification by these medications showed no significant benefit of albumin on 28-day mortality (Supplementary Table S4). Additionally, 339 patients (57.8%) in the albumin group received vasoconstrictor therapy during their ICU stay. However, regardless of vasoconstrictor therapy use, 28-day mortality did not differ significantly between patients who received albumin and those who did not (Supplementary Table S5).

### 3.5 Influence of baseline albumin and total bilirubin levels on the efficacy of albumin therapy

**FIGURE 5:**
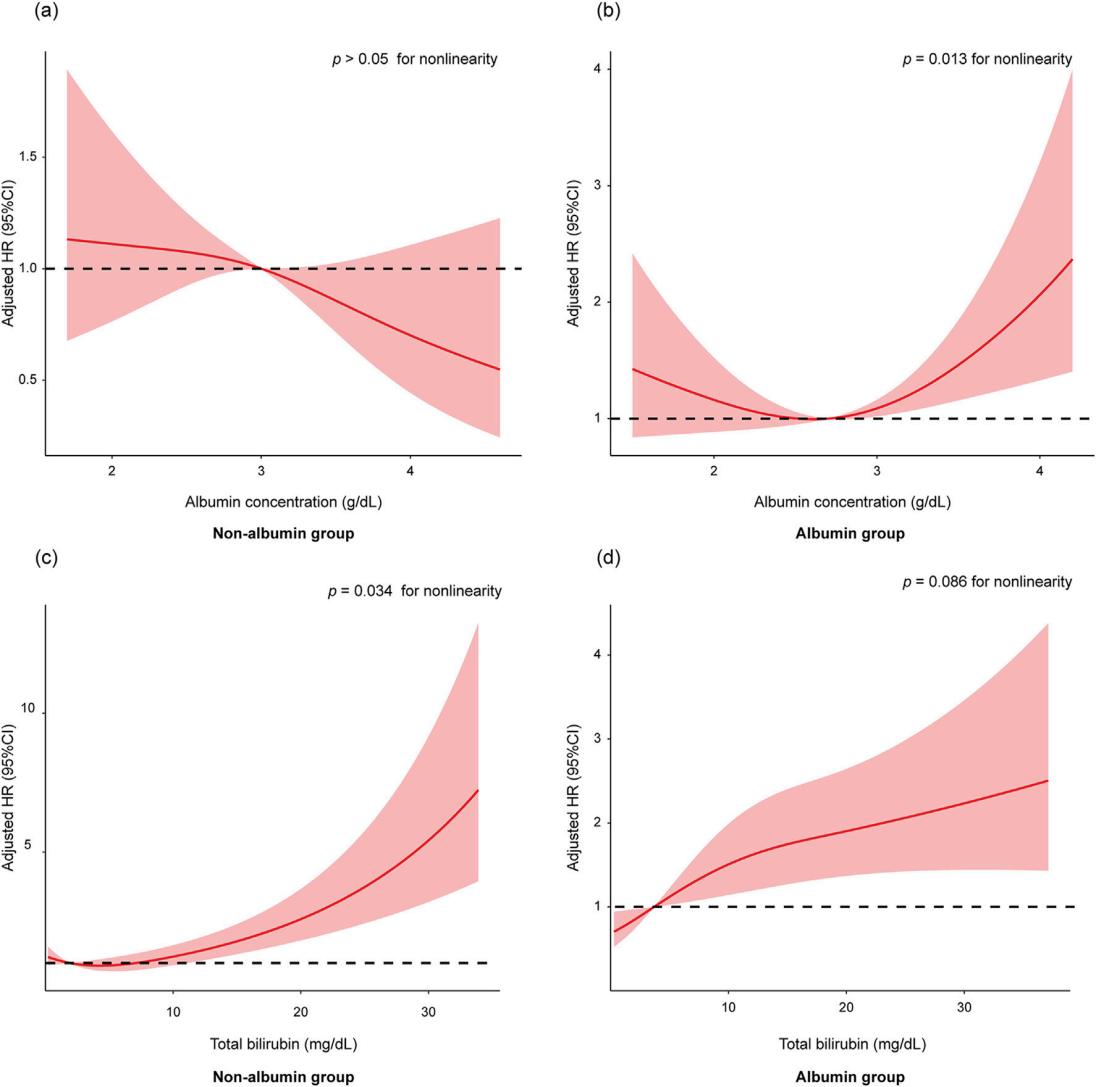
The association of baseline albumin levels with 28-day mortality in non-albumin group **(A)** and albumin group **(B)**. The association of baseline total bilirubin levels with 28-day mortality in non-albumin group **(C)** and albumin group **(D)**. The restricted cubic spline model was adjusted by age, ethnicity, total bilirubin, creatinine, urine output, INR, sodium, albumin, WBC, hemoglobin, lactate, SpO_2_, SOFA score, ascites, hepatic encephalopathy, and SBP. Abbreviations: HR, hazards ratio; INR, international normalized ratio; WBC, white blood cells; SpO_2_, peripheral capillary oxygen saturation; SOFA, Sequential Organ Failure Assessment; SBP, spontaneous bacterial peritonitis.

Next, we explored the impact of baseline albumin levels on the efficacy of albumin therapy. In the non-albumin group, baseline albumin levels were linearly associated with 28-day mortality, with higher albumin levels correlating with lower 28-day mortality (Figure 5A). In contrast, in the albumin group, baseline albumin levels exhibited a non-linear (U-shaped) relationship with 28-day mortality (Figure 5B, *p* < 0.05 for nonlinearity). We then repeated the analysis to determine the lowest serum albumin concentration at which albumin infusion still adversely affected outcomes. The results indicated that in patients with baseline albumin levels greater than 3.3 g/dL, albumin treatment significantly increased 28-day mortality compared to non-albumin treatment (Supplementary Table S6, HR 1.98; 95% CI 1.07–3.67; *p* = 0.031).

In the non-albumin treatment group, the risk of mortality increased sharply with rising total bilirubin levels (Figure 5C, *p* < 0.05 for nonlinearity). In contrast, in the albumin treatment group, the rate of increase in mortality risk was slower as total bilirubin levels rose (Figure 5D). We then repeated the analysis to identify the lowest total bilirubin levels at which albumin infusion still positively affected outcomes. The results showed that in patients with baseline total bilirubin levels greater than 15 mg/dL, albumin treatment significantly improved 28-day mortality compared to non-albumin treatment (Supplementary Table S7).

## 4 Discussion

Albumin was more likely to be administered to patients with more severe illness and greater medical complexity, as indicated by higher Model for End-Stage Liver Disease-Sodium (MELD-Na) scores, advanced stages of AKI, and increased rates of cirrhosis complications. Therefore, we used IPTW to minimize selection bias between the albumin and non-albumin groups. In the IPTW-adjusted cohort, we found that albumin administration within the first 24 h improved the 48-h kidney function recovery rate in AKI patients, indicating potential early benefits. However, this benefit did not persist for 28 days. Additionally, caution should be exercised when using albumin in patients with CKD or baseline albumin levels greater than 3.3 g/dL, due to the observed negative impact on prognosis in these subgroups.

First, we found that albumin treatment significantly increased serum albumin levels in patients, and improved renal function recovery in cirrhotic patients with AKI by 48 h after ICU admission, indicating an early benefit. Higher doses of albumin treatment led to greater increases in serum albumin levels (Supplementary Table S8). Interestingly, patients with a MELD-Na score ≥25 reached similar albumin levels by the second day as those with less severe illness (Supplementary Table S8), suggesting that higher doses may not be needed for similar results in more severely ill patients. Previous studies showed no improvement in kidney function with albumin therapy, possibly because only half of the patients received albumin within 48 h of AKI onset (Patidar et al., 2022). However, albumin administration has been associated with improved circulatory function (Fernández et al., 2005) and a significant increase in renal blood flow (Garcia-Martinez et al., 2015). These findings align with our results, where albumin use was significantly linked to AKI recovery compared to non-users.

Although albumin treatment demonstrated short-term benefits, it did not improve the 28-day mortality rate in our study. In addition to its oncotic properties, human serum albumin exhibits significant non-oncotic functions in the body, such as antioxidant activity, toxin binding and transport, and immune-modulating effects (Bernardi et al., 2020). Therefore, infusing human serum albumin may offer additional benefits beyond volume expansion by enhancing these non-oncotic functions (Baldassarre et al., 2021). Given the theoretical advantages of albumin infusion, it is likely to improve the prognosis of cirrhotic patients. However, in practical application, previous studies have shown conflicting results (Caraceni et al., 2018; Solà et al., 2018), which may be attributed to differences in patient populations, dosage, and duration of albumin therapy.

In our study focusing on ICU patients with cirrhosis and AKI, albumin therapy did not affect the 28-day mortality rate in the overall population. The short-term benefits of albumin therapy did not appear to persist at 28 days. Survival at 28 days was primarily influenced by the patient’s underlying disease state, with factors such as age, total bilirubin, urine output, INR, and sodium levels proving significant in the multivariable Cox regression analysis (Supplementary Table S9). Previous studies have reported similar findings, showing that albumin therapy improved the 7-day survival rate in cirrhosis patients with sepsis (Philips et al., 2021), but did not lead to improved 28-day survival rates (Maiwall et al., 2022). This may be due to the lower proportion of effective albumin in commercial albumin preparations compared to that in cirrhotic patients (Baldassarre et al., 2021). The low proportion of effective albumin could result in the benefits brought by its non-oncotic properties lacking clinical significance. Additionally, the half-life of albumin is approximately 15 days (Quinlan et al., 2005). In pathological conditions, increased albumin degradation and enhanced transcapillary leakage lead to an accelerated decrease in albumin concentration after infusion (Keller, 2019), potentially limiting its long-term beneficial effects.

Additionally, ICU patients constitute a distinct population characterized by severe illness and multiple comorbidities, which could potentially increase the risks associated with albumin administration. Other studies have found that lower partial pressure of oxygen, pneumonia, and sicker patients with higher MELD, Sequential Organ Failure Assessment (SOFA) scores, and arterial lactate levels are risk factors for pulmonary complications (Maiwall et al., 2022). This could also explain why the 28-day mortality rate did not improve in the albumin treatment group in our study. However, drug-related adverse reactions were not recorded in this cohort, thus further studies are needed to validate these findings. Our study also showed that central venous pressure increased more noticeably in the albumin group, although this difference was not statistically significant (Supplementary Table S10). Due to the invasive nature of central venous pressure measurement, many values were missing. In the future, non-invasive methods like the Venous Excess Ultrasound score or measuring the diameter of the inferior vena cava should be considered for monitoring blood volume status and preventing potential complications (Banegas-Deras et al., 2024).

Second, in subgroup analyses, we found that albumin therapy did not improve the 28-day mortality rate regardless of AKI severity, or the presence of various comorbidities and complications. However, it is noteworthy that albumin use had a negative effect on patients with a history of CKD (Figure 4, *p* < 0.05). In our cohort, 353 patients had CKD, with 188 (53.3%) attributed to hypertension, 45 (12.7%) to diabetes, and 30 (8.5%) to both diabetes and hypertension. The cause for the remaining patients is unknown. The negative effect of albumin in CKD patients may be due to CKD patients often experiencing chronic volume overload (Zoccali et al., 2023). Specifically, those with CKD caused by hypertension or diabetes frequently have severe vascular damage and fluid management issues, which can be exacerbated by albumin administration, leading to a deterioration in their overall clinical condition. However, albumin had no significant effect on AKI recovery, regardless of the presence of CKD (Supplementary Table S11). These findings suggest that the decision to use albumin should be carefully weighed in critically ill patients, particularly those with CKD, due to potential side effects and economic costs.

Additionally, using a 5% albumin concentration seems to improve the 28-day mortality rate. Although the guidelines recommend the use of 20% albumin (European Association for the Study of the LiverEuropean Association for the Study of the Liver, 2018), this may be because, in the ICU population, the volume expansion effect of 5% albumin is more moderate compared to 20% albumin (Jardot et al., 2024), making it safer for ICU patients with multiple comorbidities. While the use of 5% albumin shows promise in improving 28-day mortality, further prospective studies are needed to confirm this finding.

Third, previous studies have shown that higher albumin levels often correlate with a better prognosis (Ripoll et al., 2015). However, albumin therapy seemed to modify this relationship. In the albumin treatment group, a U-shaped relationship was observed between baseline albumin levels and 28-day mortality (Figure 5B). Administering albumin to patients with higher baseline albumin levels may suppress their own albumin synthesis (Yuan et al., 2008). Furthermore, we found that in patients with albumin levels greater than 3.3 g/dL, albumin treatment was associated with increased 28-day mortality compared to the non-albumin treatment group (Supplementary Table S6, *p* < 0.05). In patients with baseline serum albumin ≤3.3 g/dL, albumin treatment improved AKI regression and recovery compared to no albumin (Supplementary Table S12, *p* < 0.05), but this effect was not observed in those with baseline albumin >3.3 g/dL (Supplementary Table S12). The potential side effects of albumin may counterbalance its benefits on AKI recovery in patients with baseline albumin ≤3.3 g/dL, leading to no significant impact on 28-day mortality. However, in patients with baseline albumin >3.3 g/dL, the lack of improvement in AKI recovery combined with albumin’s side effects might have contributed to a higher 28-day mortality. Therefore, our research suggests that albumin treatment for patients with higher baseline albumin levels, especially those greater than 3.3 g/dL, should be approached with greater caution.

Cholemic nephropathy, an often overlooked cause of AKI in liver diseases, is associated with varying degrees of cholestasis (Krones et al., 2018; Fickert and Rosenkranz, 2020; Fickert, 2024; Krones et al., 2017). Since the diagnosis relies on biopsy, it often goes unrecognized in clinical practice (Ghallab et al., 2024). Due to the lack of relevant total bile acid data, we grouped patients based on their bilirubin levels. As shown in Supplementary Table S7, the results indicated that in patients with baseline total bilirubin levels greater than 15 mg/dL, albumin treatment significantly improved 28-day mortality compared to non-albumin treatment. However, no significant effect on AKI recovery was observed in patients with total bilirubin >15 mg/dL (Supplementary Table S13). This suggests that albumin therapy may not effectively reverse kidney damage caused by bile acid overload. The improvement in 28-day mortality in the high bilirubin group might be related to albumin’s ability to improve endothelial function and bind toxic substances. Future studies should evaluate the efficacy of albumin therapy under different bile acid loads, particularly in patients with confirmed cholemic nephropathy.

This study has several limitations. Firstly, it utilizes patient data from the MIMIC-IV database, which is a single-center retrospective cohort. The decision to use albumin therapy was not standardized and likely varied among physicians, potentially introducing selection bias. We have attempted to minimize this bias through the application of IPTW methods. Secondly, the dosage and duration of albumin therapy were not standardized. Since this is a retrospective study, we could not determine the specific reasons for the varying doses and treatment durations received by patients. Additionally, in this study, the albumin dosage was relatively low, and the duration of treatment was relatively short. However, these data reflect the use of albumin in real-world clinical settings. Furthermore, even in subgroup analyses with higher doses and longer durations of albumin use, the results remained unchanged (Table 2). Despite these limitations, given the current lack of randomized controlled trials focusing on cirrhotic patients with AKI, our study still offers valuable clinical insights, especially for ICU populations. Future randomized controlled trials studies are warranted to further investigate this area.

In this study, albumin infusion improved AKI recovery within the first 48 h of ICU admission in the overall population but was not associated with 28-day mortality. However, albumin therapy may be harmful to certain subgroups, such as patients with CKD and higher baseline albumin levels. Conversely, in patients with high bilirubin levels, albumin treatment significantly improved 28-day mortality. Based on these findings, the use of albumin in critically ill patients with cirrhosis and AKI should be approached with greater consideration of its risks and benefits, exercising more caution in clinical practice.

## Data Availability

The data supporting the findings of this study are available from the corresponding author upon reasonable request.
