# Supplementary material for: Impact of albumin infusion on prognosis in ICU patients with cirrhosis and AKI: insights from the MIMIC-IV database
Source: Front Pharmacol. 2024 Oct 7;15:1467752. doi: 10.3389/fphar.2024.1467752 (PMC11491358; doi:10.3389/fphar.2024.1467752)
Supplement: Supplementary file 1 [file Table1.docx]

**Supplementary Table 1** Diagnosis codes from ICD-9 and ICD-10.

| **Variable** | **ICD-9 Codes** | **ICD-10 Codes** |
| --- | --- | --- |
| Cirrhosis | 5712, 5715, 5716 | K703, K7030, K7031, K741, K742, K743, K744, K745, K746, K7460, K7469 |
| Hepatitis C | 0700, 0701, 07020, 07021, 07022, 07023, 07030, 07031, 07032, 07033, 07041, 07042, 07043, 07044, 07049, 07051, 07052, 07053, 07054, 07059, 0706, 07070, 07071, 0709 | B15, B150, B159, B16, B160, B161, B162, B169, B17, B170, B171, B1710, B1711, B172, B178, B179, B18, B180, B181, B182, B188, B189, B19, B190, B191, B1910, B1911, B192, B1920, B1921, B199 |
| Alcohol | 5712 | K703, K7030, K7031 |
| Ascites | 78959 | K7011, K7031, K7151, R188 |
| Esophageal varices with bleeding | 4560, 45620 | I8501, I8511 |
| Hepatic encephalopathy | 0700, 07020, 07021, 07022, 07023, 07041, 07042, 07043, 07044, 07049, 0706, 07071, 5722 | B150, B160, B162, B1711, B190, B1911, B1921, K7201, K7211, K7291, K7041, K7111 |
| Spontaneous bacterial peritonitis | 56723 | K652 |
| CKD | 28521, 40300, 40301, 40310, 40311, 40390, 40391, 40400, 40401, 40402, 40403, 40410, 40411, 40412, 40413, 40490, 40491, 40492, 40493, 5851, 5852, 5853, 5854, 5855, 5859 | D631, E0822, E0922, E1022, E1122, E1322, I12, I120, I129, I13, I130, I131, I1310, I1311, I132, N18, N181, N182, N183, N184, N185, N189, O102, O1021, O10211, O10212, O10213, O10219, O1022, O1023, O103, O1031, O10311, O10312, O10313, O10319, O1032, O1033 |

Abbreviations: ICD, International Classification of Diseases; CKD, chronic kidney disease.

**Supplementary Table 2** Missing rate for demographics and clinical variables extracted from the database on the first day.

| Variable | Number of missing | Percent of missing (%) |
| --- | --- | --- |
| Age | 0 | 0 |
| Gender | 0 | 0 |
| Ethnicity | 0 | 0 |
| Aetiology of cirrhosis | 0 | 0 |
| Stage of AKI | 0 | 0 |
| **Severity of illness** |  |  |
| MELD-Na score | 0 | 0 |
| SOFA score | 2 | 0.12 |
| GCS | 4 | 0.25 |
| Charlson comorbidity index | 0 | 0 |
| **Laboratory measurements** |  |  |
| ALT | 311 | 19.16 |
| AST | 308 | 18.98 |
| ALP | 311 | 19.16 |
| Creatinine | 175 | 10.78 |
| BUN | 174 | 10.72 |
| Bicarbonate | 175 | 10.78 |
| Albumin | 307 | 18.92 |
| Total bilirubin | 309 | 19.04 |
| Chloride | 174 | 10.72 |
| Sodium | 174 | 10.72 |
| Potassium | 174 | 10.72 |
| WBC | 174 | 10.72 |
| Hemoglobin | 174 | 10.72 |
| Platelet | 175 | 10.78 |
| INR | 202 | 12.45 |
| Lactate | 264 | 16.27 |
| SpO2 | 2 | 0.12 |
| **Vital signs** |  |  |
| Heart rate | 3 | 0.18 |
| MAP | 3 | 0.18 |
| RR | 2 | 0.12 |
| Temperature | 34 | 2.09 |
| **Complications or comorbidities** |  |  |
| Esophageal varices with bleeding | 0 | 0 |
| Spontaneous bacterial peritonitis | 0 | 0 |
| Hepatic encephalopathy | 0 | 0 |
| Ascites | 0 | 0 |
| Bacterial infections | 0 | 0 |
| Septic | 0 | 0 |
| Shock | 0 | 0 |
| CKD | 0 | 0 |
| **Treatments within 24h after ICU admission** |  |  |
| RRT | 0 | 0 |
| Diuretics | 0 | 0 |
| NSBB | 0 | 0 |
| Mechanical ventilation | 0 | 0 |
| Urine output | 53 | 3.27 |

Abbreviations: AKI, acute kidney injury; MELD-Na, Model for End-Stage Liver Disease-Sodium; SOFA, Sequential Organ Failure Assessment; GCS, Glasgow Coma Scale; ALT, alanine aminotransferase; AST, aspartate aminotransferase; ALP, alkaline phosphatase; BUN, blood urea nitrogen; WBC, white blood cells; INR, international normalized ratio; SpO_2_, peripheral capillary oxygen saturation; MAP, mean arterial pressure; RR, respiration rate; CKD, chronic kidney disease; RRT, renal replacement therapy; NSBB, non-selective beta blocker.

**Supplementary Table 3** Comparison of albumin levels on day 1 and day 2 after ICU admission

| Group | Albumin (g/dL)  Day 1 | Albumin (g/dL)  Day 2 | *P* value |
| --- | --- | --- | --- |
| Non-albumin group (n=1037) ^a^ | 2.9 (2.6-3.3) | 2.8 (2.5-3.2) | <0.001 |
| Albumin group (n=586) ^b^ | 2.6 (2.3-3.0) | 3.1 (2.7-3.5) | <0.001 |

Abbreviations: ICU, intensive care unit.

^a^ A total of 331 patients had both albumin levels available on Day 1 and Day 2 in non-albumin group. ^b^ A total of 248 patients had both albumin levels available on Day 1 and Day 2 in albumin group.

**Supplemental Table 4** Association between albumin treatment and 28-day mortality stratified by the use of diuretics or nephrotoxic drugs

|  | Univariable model | | Multivariable model | | IPTW | |
| --- | --- | --- | --- | --- | --- | --- |
|  | HR (95% CI) | *P* value | HR (95% CI) | *P* value | HR (95% CI) | *P* value |
| **Diuretic** |  |  |  |  |  |  |
| Yes (n=382) | 1.65 (1.11-2.45) | 0.013 | 0.80 (0.49-1.31) | 0.375 | 0.66 (0.35-1.26) | 0.207 |
| No (n=1241) | 2.33 (1.84-2.96) | <0.001 | 1.11 (0.84-1.47) | 0.456 | 0.99 (0.66-1.49) | 0.950 |
| **NSBB** |  |  |  |  |  |  |
| Yes (n=95) | 2.36 (0.79-7.02) | 0.123 | 0.14 (0.01-2.54) | 0.185 | 1.35 (0.40-4.60) | 0.630 |
| No (n=1528) | 2.10 (1.70-2.58) | <0.001 | 0.99 (0.78-1.25) | 0.900 | 1.01 (0.74-1.37) | 0.971 |
| **NSAIDs** |  |  |  |  |  |  |
| Yes (n=195) | 1.03 (0.50-2.16) | 0.929 | 0.40 (0.14-1.12) | 0.080 | 0.56 (0.22-1.43) | 0.224 |
| No (n=1428) | 2.27 (1.83-2.81) | <0.001 | 1.06 (0.83-1.36) | 0.640 | 0.99 (0.68-1.43) | 0.945 |
| **Aminoglycosides** |  |  |  |  |  |  |
| Yes (n=76) | 1.40 (0.70-2.82) | 0.343 | 0.39 (0.12-1.25) | 0.114 | 1.40 (0.71-2.78) | 0.332 |
| No (n=1547) | 2.23 (1.80-2.76) | <0.001 | 0.97 (0.75-1.24) | 0.781 | 0.96 (0.69-1.35) | 0.822 |

Multivariate model was adjusted for age, ethnicity, total bilirubin, creatinine, urine output, INR, sodium, albumin, WBC, hemoglobin, lactate, SpO_2_, SOFA score, ascites, hepatic encephalopathy, and SBP.

Abbreviations: IPTW, inverse probability of treatment weighting; HR, hazard ratio; NSBB, non-selective β-blockers; NSAIDs, non-steroidal anti-inflammatory drugs; INR, international normalized ratio; WBC, white blood cells; SpO_2_, peripheral capillary oxygen saturation; SOFA, Sequential Organ Failure Assessment; SBP, spontaneous bacterial peritonitis.

**Supplementary Table 5** Association between albumin treatment and 28-day mortality stratified by vasoconstrictor use

| Subgroup | Univariable model | | Multivariable model | |
| --- | --- | --- | --- | --- |
|  | HR (95% CI) | *P* value | HR (95% CI) | *P* value |
| Non-albumin treatment (n=1037) | Ref |  | Ref |  |
| Albumin without vasoconstrictor (n=247) | 1.20 (0.88-1.64) | 0.248 | 0.90 (0.65-1.26) | 0.540 |
| Albumin with vasoconstrictor (n=339) | 2.94 (2.36-3.67) | <0.001 | 1.01 (0.78-1.31) | 0.939 |

Multivariate model was adjusted for age, ethnicity, total bilirubin, creatinine, urine output, INR, sodium, albumin, WBC, hemoglobin, lactate, SpO_2_, SOFA score, ascites, hepatic encephalopathy, and SBP.

Abbreviations: HR, hazard ratio; INR, international normalized ratio; WBC, white blood cells; SpO_2_, peripheral capillary oxygen saturation; SOFA, Sequential Organ Failure Assessment; SBP, spontaneous bacterial peritonitis.

**Supplementary Table 6** Multivariable Cox mode for 28-day mortality grouped by baseline albumin concentration.

The analysis was repeated until reaching the lowest level of serum albumin concentration, for which the albumin infusion still showed a negative effect on the outcome.

| Serum albumin concentration cut-off | Univariable model | | Multivariable model | |
| --- | --- | --- | --- | --- |
|  | HR (95% CI) | *P* value | HR (95% CI) | *P* value |
| ≤3.3 g/dL (n=1231) | 1.73 (1.37-2.17) | <0.001 | 0.84 (0.65-1.09) | 0.184 |
| >3.3 g/dL (n=392) | 4.53 (2.85-7.18) | <0.001 | 1.98 (1.07-3.67) | 0.031 |

The multivariate model was adjusted for age, ethnicity, total bilirubin, creatinine, urine output, INR, sodium, albumin, WBC, hemoglobin, lactate, SpO_2_, SOFA score, ascites, hepatic encephalopathy, and SBP.

Abbreviations: HR, hazard ratio; INR, international normalized ratio; WBC, white blood cells; SpO_2_, peripheral capillary oxygen saturation; SOFA, Sequential Organ Failure Assessment; SBP, spontaneous bacterial peritonitis.

**Supplementary Table 7** Multivariable Cox mode for 28-day mortality grouped by baseline total bilirubin.

|  | Univariable model | | Multivariable model | |
| --- | --- | --- | --- | --- |
|  | HR (95% CI) | *P* value | HR (95% CI) | *P* value |
| Total bilirubin (mg/dL) |  |  |  |  |
| ≤15 (n=1231) | 2.03 (1.62-2.56) | <0.001 | 1.19 (0.91-1.54) | 0.204 |
| >15 (n=132) | 1.20 (0.75-1.93) | 0.448 | 0.53 (0.30-0.97) | 0.040 |

The analysis was repeated until reaching the lowest level of total bilirubin, for which the albumin infusion still showed a positive effect on the outcome.

The multivariate model was adjusted for age, ethnicity, total bilirubin, creatinine, urine output, INR, sodium, albumin, WBC, hemoglobin, lactate, SpO_2_, SOFA score, ascites, hepatic encephalopathy, and SBP.

Abbreviations: HR, hazard ratio; INR, international normalized ratio; WBC, white blood cells; SpO_2_, peripheral capillary oxygen saturation; SOFA, Sequential Organ Failure Assessment; SBP, spontaneous bacterial peritonitis.

**Supplementary Table 8** Comparison of albumin levels on day 1 and day 2 after ICU admission in patients receiving different doses of albumin

| Group^a^ | Albumin (g/dL)  Day 1 | Albumin (g/dL)  Day 2 | *P* value |
| --- | --- | --- | --- |
| **Total Population** |  |  |  |
| < 1.0 g/kg/day (n=169) | 2.7 (2.4-3.0) | 2.9 (2.6-3.2) | <0.001 |
| ≥ 1.0 g/kg/day (n=79) | 2.6 (2.3-3.1) | 3.3 (3.1-3.8) | <0.001 |
| **MELD-Na score ≥ 25** |  |  |  |
| < 1.0 g/kg/day (n=95) | 2.6 (2.3-3.0) | 2.9 (2.6-3.3) | <0.001 |
| ≥ 1.0 g/kg/day (n=45) | 2.6 (2.2-3.1) | 3.3 (3.1-4.0) | <0.001 |
| **MELD-Na score < 25** |  |  |  |
| < 1.0 g/kg/day (n=74) | 2.7 (2.4-3.0) | 2.9 (2.7-3.2) | <0.001 |
| ≥ 1.0 g/kg/day (n=34) | 2.7 (2.3-3.0) | 3.4 (3.0-3.8) | <0.001 |

Abbreviations: ICU, intensive care unit; MELD-Na, Model for End-Stage Liver Disease-Sodium.

^a^ A total of 248 patients had both albumin levels available on Day 1 and Day 2 in albumin group.

**Supplementary Table 9** Risk factors associated with 28-day mortality in cirrhosis patients with AKI.

|  | Univariable model | | Multivariable model | |
| --- | --- | --- | --- | --- |
|  | HR (95% CI) | *P* value | HR (95% CI) | *P* value |
| Albumin treatment | 2.13 (1.73-2.61) | <0.001 | 0.97 (0.77-1.23) | 0.824 |
| Age, years | 1.02 (1.01-1.03) | <0.001 | 1.04 (1.03-1.05) | <0.001 |
| Gender |  |  |  |  |
| Male | Ref |  | - | - |
| Female | 0.94 (0.75-1.17) | 0.561 | - | - |
| Ethnicity |  |  |  |  |
| White | Ref |  | Ref |  |
| Black | 0.84 (0.56-1.27) | 0.402 | 0.80 (0.52-1.23) | 0.301 |
| Asian | 1.69 (0.97-2.95) | 0.066 | 0.70 (0.36-1.35) | 0.282 |
| Other | 1.26 (0.99-1.60) | 0.058 | 0.89 (0.70-1.15) | 0.385 |
| Total bilirubin, mg/dL | 1.06 (1.05-1.07) | <0.001 | 1.04 (1.03-1.05) | <0.001 |
| Creatinine, mg/dL | 1.10 (1.06-1.13) | <0.001 | 0.95 (0.89-1.01) | 0.107 |
| Urine output, L | 0.48 (0.41-0.56) | <0.001 | 0.77 (0.67-0.89) | <0.001 |
| INR | 1.22 (1.17-1.27) | <0.001 | 1.12 (1.05-1.20) | 0.001 |
| Sodium, mmol/L | 0.97 (0.96-0.99) | <0.001 | 0.98 (0.96-1.00) | 0.023 |
| Albumin, g/dL | 0.69 (0.59-0.81) | <0.001 | 1.00 (0.85-1.17) | 0.981 |
| WBC, 10^9^/L | 1.03 (1.03-1.04) | <0.001 | 1.02 (1.01-1.03) | <0.001 |
| Hemoglobin, g/dL | 0.90 (0.86-0.95) | <0.001 | 0.99 (0.94-1.04) | 0.630 |
| Lactate, mmol/L | 1.15 (1.13-1.17) | <0.001 | 1.07 (1.04-1.09) | <0.001 |
| SpO_2_, mmHg | 0.96 (0.96-0.97) | <0.001 | 0.99 (0.98-0.99) | 0.001 |
| SOFA score | 1.25 (1.22-1.28) | <0.001 | 1.17 (1.13-1.20) | <0.001 |
| Ascites | 1.47 (1.16-1.86) | 0.001 | 0.96 (0.73-1.25) | 0.735 |
| Esophageal varices with bleeding | 0.97 (0.68-1.38) | 0.860 | - | - |
| Hepatic encephalopathy | 1.37 (1.07-1.76) | 0.014 | 0.89 (0.67-1.18) | 0.405 |
| Spontaneous bacterial peritonitis | 1.98 (1.44-2.71) | <0.001 | 1.16 (0.83-1.62) | 0.390 |

The multivariate model was adjusted for age, ethnicity, total bilirubin, creatinine, urine output, INR, sodium, albumin, WBC, hemoglobin, lactate, SpO_2_, SOFA score, ascites, hepatic encephalopathy, and SBP.

Abbreviations: AKI, acute kidney injury; HR, hazard ratio; INR, international normalized ratio; WBC, white blood cells; SpO_2_, peripheral capillary oxygen saturation; SOFA, Sequential Organ Failure Assessment; SBP, spontaneous bacterial peritonitis.

**Supplementary Table 10** Comparison of CVP on Day 1 and Day 2

| Group | CVP (mmHg)  Day 1 | CVP (mmHg)  Day 2 | *P* value |
| --- | --- | --- | --- |
| Non-albumin group (n=1037) ^a^ | 12.3 (9.0-17.2) | 11.6 (8.4-17.0) | 0.313 |
| Albumin group (n=586) ^b^ | 11.1 (7.8-15.5) | 11.5 (7.0-17.6) | 0.082 |

^a^ A total of 126 patients had both CVP measurements available on Day 1 and Day 2 in non-albumin group. ^b^ A total of 164 patients had both CVP measurements available on Day 1 and Day 2 in album in group.

Abbreviations: CVP, central venous pressure.

**Supplementary Table 11** Comparison of AKI regression and full recovery rates between albumin and non-albumin groups stratified by with or without CKD

|  | Non-albumin group | Albumin group | *P* value |
| --- | --- | --- | --- |
| **With CKD ^a^** |  |  |  |
| Regression of AKI, n (%) | 78 (22.0) | 101 (29.8) | 0.181 |
| Full recovery of AKI, n (%) | 61 (17.2) | 57 (16.8) | 0.966 |
| **Without CKD ^b^** |  |  |  |
| Regression of AKI, n (%) | 407 (30.8) | 414 (34.0) | 0.389 |
| Full recovery of AKI, n (%) | 314 (23.7) | 331 (27.2) | 0.301 |

Weights based on propensity scores were recalculated for each subgroup analysis.

^a^ In the IPTW cohort, non-albumin group (n = 354), albumin group (n = 339)

^b^ In the IPTW cohort, non-albumin group (n = 1323), albumin group (n = 1216)

Abbreviations: AKI, acute kidney injury; CKD, chronic kidney disease; IPTW, inverse probability of treatment weighting.

**Supplementary Table 12** Comparison of AKI regression and full recovery rates between albumin and non-albumin groups stratified by albumin levels

| Serum albumin | Non-albumin group | Albumin group | *P* value |
| --- | --- | --- | --- |
| **≤3.3 g/dL ^a^** |  |  |  |
| Regression of AKI | 340 (26.5) | 417 (35.5) | 0.009 |
| Full recovery of AKI | 258 (20.1) | 340 (28.9) | 0.005 |
| **>3.3 g/dL ^b^** |  |  |  |
| Regression of AKI | 188 (25.0) | 212 (29.9) | 0.360 |
| Full recovery of AKI | 143 (19.0) | 156 (22.0) | 0.564 |

Weights based on propensity scores were recalculated for each subgroup analysis.

^a^ In the IPTW cohort, non-albumin group (n=1282)，albumin group (n=1175)

^b^ In the IPTW cohort, non-albumin group (n=752)，albumin group (n=710)

Abbreviations: AKI, acute kidney injury; IPTW, inverse probability of treatment weighting.

**Supplementary Table 13** Comparison of AKI regression and full recovery rates between albumin and non-albumin groups stratified by total bilirubin levels

| Total bilirubin | Non-albumin group | Albumin group | *P* value |
| --- | --- | --- | --- |
| **≤15 mg/dL ^a^** |  |  |  |
| Regression of AKI | 340 (22.1) | 393 (28.9) | 0.041 |
| Full recovery of AKI | 438 (28.5) | 492 (36.1) | 0.030 |
| **>15 mg/dL ^b^** |  |  |  |
| Regression of AKI | 31 (25.0) | 23 (19.2) | 0.481 |
| Full recovery of AKI | 23 (18.5) | 12 (10.0) | 0.218 |

Weights based on propensity scores were recalculated for each subgroup analysis.

^a^ In the IPTW cohort, non-albumin group (n=1535)，albumin group (n=1362)

^b^ In the IPTW cohort, non-albumin group (n=124)，albumin group (n=120)

Abbreviations: AKI, acute kidney injury; IPTW, inverse probability of treatment weighting.
